# The analgesic contribution of external oblique intercostal block: Case reports of 3 different surgeries and 3 spectacular effects

**DOI:** 10.1097/MD.0000000000030435

**Published:** 2022-09-09

**Authors:** Sami Kaan Coşarcan, Ömür Erçelen

**Affiliations:** a VKV American Hospital, Anesthesiology, Istanbul, Turkey; b VKV American Hospital, Anesthesiology and Pain, Istanbul, Turkey.

**Keywords:** bariatric surgery, external oblique intercostal block, fascial plane blocks, liver surgery

## Abstract

Abdominal wall blocks are frequently used due to the use of effective blocks, such as the transversus abdominis plane (TAP) block and the widespread use of ultrasound (US) imaging. A good knowledge of abdominal innervation is required for the use of abdominal wall blocks. We describe the extraordinary performance of external oblique intercostal (EOI) blocks in 3 different surgeries.

**Patient concerns, diagnosis and interventions:**

Case 1: A man aged 30 to 35 was taken to the operating room for open liver surgery. After surgery, unilateral EOI block and bilateral TAP block were performed with the patient in the supine position, and a catheter was placed under the external oblique muscle. Postoperative analgesia was followed by patient-controlled analgesia (PCA) through the catheter. Case 2: A male patient aged 35 to 40 was taken to the operating room for laparoscopic liver surgery. After surgery, unilateral (EOI) block and bilateral TAP block were performed with the patient in the supine position. The patient received iv tramadol PCA (bolus dose 10 mg only, lockout 20 minutes). Case 3: A man aged 25 to 30 was taken to the operating room for laparoscopic bariatric surgery. After the surgery, bilateral EOI and bilateral rectal sheath blocks were performed with the patient in the supine position. The patient received iv tramadol PCA (bolus dose 10 mg only, lockout 20 minutes).

**Outcomes:**

All patients had low NRS scores in the recovery unit and very low opioid consumption in the first 72 hours postoperatively. All were satisfied with the quality of analgesia.

**Conclusion:**

We think that EOI block will come to occupy a significant place in upper abdominal analgesia, especially in obese patients, due to its wide innervation area and ease of application.

**Key points**

• External oblique intercostal block may be the first choice in bariatric surgery because it is easy to apply in patients with obesity and has effective analgesia.
• The application site can be easily visualized independently of body mass index and is easily accessible, and it also represents no disadvantage for obese patients under USG.
• EOI block shows sufficient analgesic effect in upper abdominal surgery.
• EOI block is also suitable for the use of catheters.

## 1. Introduction

Regional anesthesia of the trunk and abdominal wall is usually centered on epidural analgesia. However, with the increasing use of minimally invasive laparoscopic techniques, the importance of postoperative anticoagulation regimens and early ambulation has led to changes in the choice of analgesia. The popularity of abdominal wall blocks has increased dramatically in recent years. These are frequently used due to the use of blocks that are highly effective, such as the transversus abdominis plane (TAP) block and the widespread use of ultrasound (US) imaging.^[1]^ However, a good knowledge of abdominal innervation is required for the use of abdominal wall blocks.

The anterior abdominal wall is innervated by the thoracoabdominal nerves and the ilioinguinal and iliohypogastric nerves. The thoracoabdominal nerves originate from the anterior rami of the T6-T12 nerves. These each give off a lateral cutaneous branch in the midaxillary line. The anterior branches of the thoracoabdominal nerves travel in the neurovascular TAP between the internal oblique muscle and transversus abdominis muscle. The terminal anterior cutaneous branches of the thoracoabdominal nerves enter the rectus muscle sheath at the linea semilunaris.^[1,2]^

Although TAP block varies depending on the different approaches adopted, it blocks the T7-L1 spinal nerves. Subcostal oblique TAP block innervation occurs at the T6-T9 levels. Various quadratus lumborum blocks target at the T7-L1 level. The innervation of the rectus sheath block (RB) may be limited to the midline.^[3,4]^ Depending on the surgery to be performed, the anticipated pain potential should be well resolved, and a fascial plane block or combinations should be considered accordingly.

EOI block is performed by placing the US probe in paramedian sagittal orientation at the T6-7 level and visualizing the external oblique and intercostal muscles. Local anesthetics inject the under the external oblique muscle.

The application of TAP block and RB can be difficult, especially in obese patients. In addition, even if performed bilaterally, TAP block may not provide full analgesia in the abdominal wall. The EOI block can represent a particularly important option from that perspective. We think that it will come to occupy an important place in terms of both ease of application and a wide analgesia area in the upper abdomen. This report describes the extraordinary performance of EOI block in 3 different surgeries.

## 2. Case report

### 2.1. Case 1

**Figure 1.**
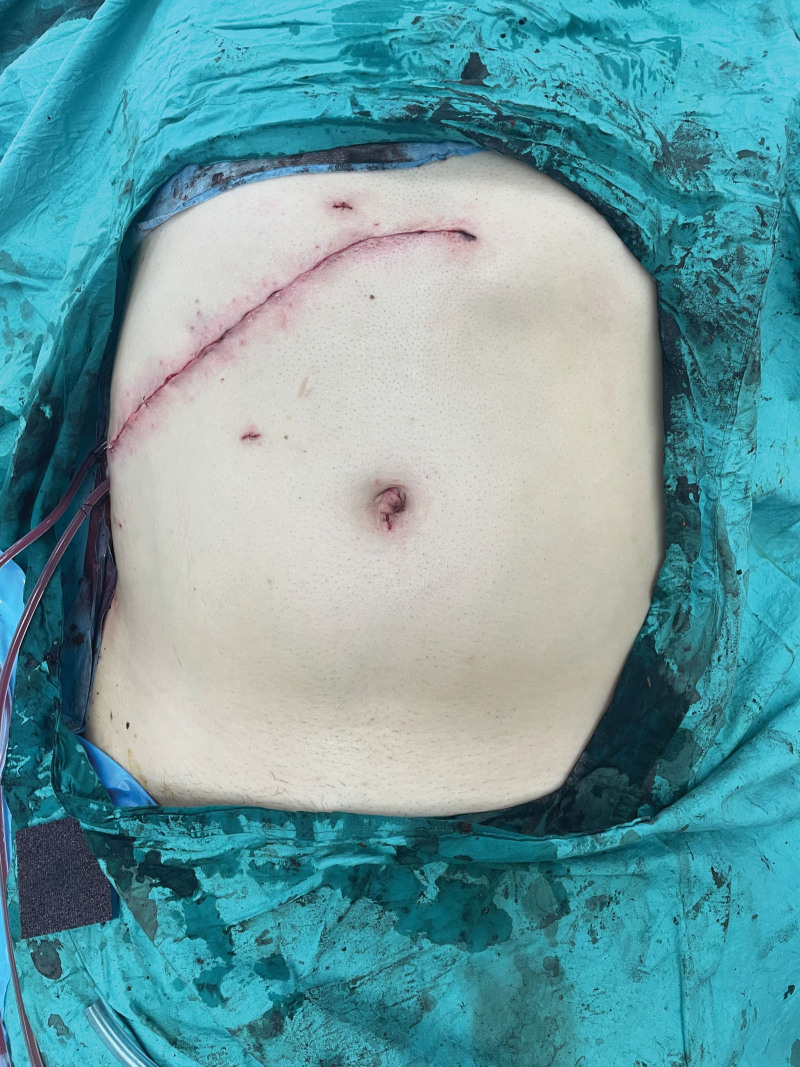
Kocher incision for open liver surgery.

**Figure 2.**
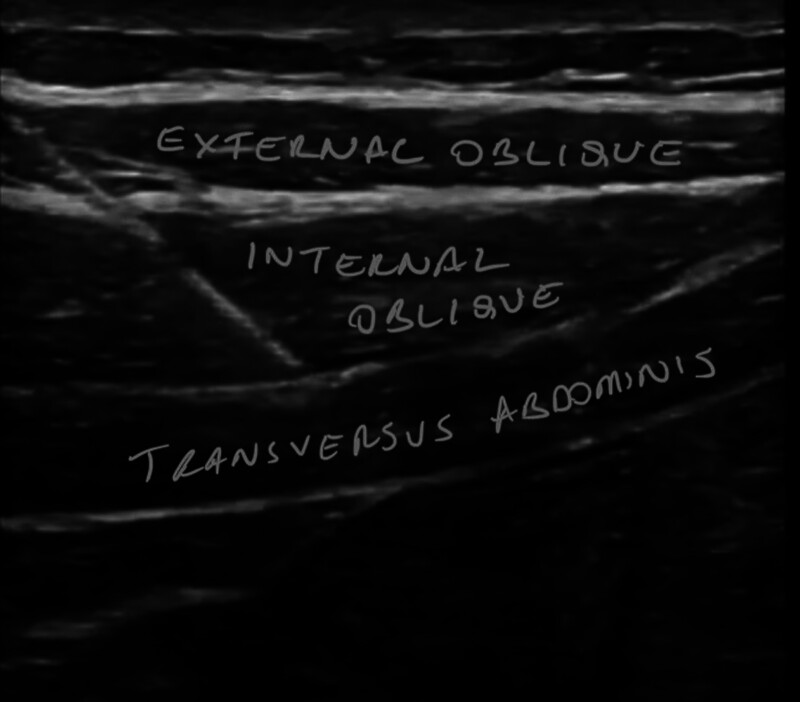
Ultrasound-guided transversus abdominis block and needle placement.

**Figure 3.**
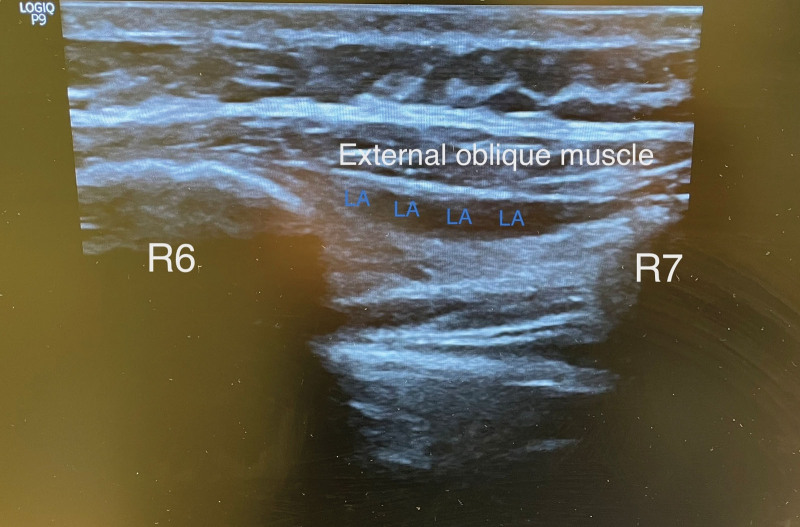
Ultrasound-guided external oblique intercostal block—local anesthetic distribution between the external oblique and intercostal muscles.

**Figure 4.**
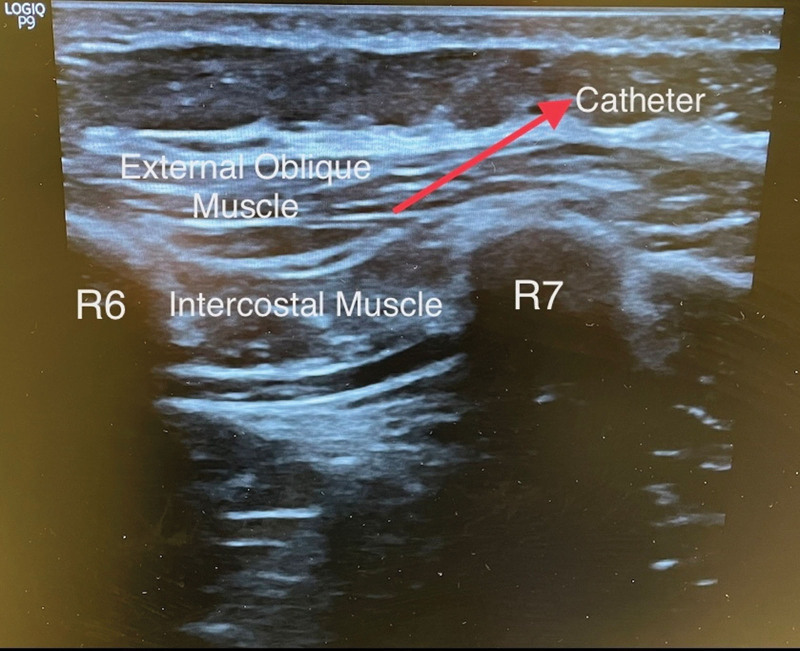
Catheter placement for EOI block. EOI = external oblique intercostal.

A man aged 30 to 35 was taken to the operating room for laparoscopic liver surgery. General anesthesia was performed with 2 mg·kg^− 1^ propofol, 1 μg·kg^−1^ fentanyl, and 0.6 mg·kg^−1^ rocuronium. Anesthesia was maintained by remifentanil infusion and desflurane. The operation commenced laparoscopically and developed into an open procedure through a right upper quadrant Kocher incision (Fig. 1) due to severe adhesions and a difficult anatomical structure. Segmentectomy + choledectomy and cholecystectomy was performed. Operative time was 9 hours. After the surgery, EOI and bilateral TAP block were performed after surgery with the patient in the supine position. Blocks were performed under US guidance (GE, LOGIQ P9 R3, Seongnam-si, Republic of Korea) with a large bandwidth, multifrequency linear probe (4–12MHz), and a 22G, 50 mm, insulated facet type needle (Braun Sonoplex, Melsungen, Germany). TAP block for each side consisted of 20 mL of subcostal 0.25% bupivacaine at the anterior axillary level between the internal oblique and transversus abdominis muscle (Fig. 2). EOI block on the right side involved 20 mL of 0.25% bupivacaine from the sixth rib level between the external oblique and intercostal muscles (Fig. 3), and the catheter was placed under the external oblique muscle (Fig. 4). The patient was extubated after the block. At the end of surgery, the patient received 1 g paracetamol and tramadol 50 mg iv. Postoperative analgesia was followed by patient-controlled analgesia (PCA) through the catheter. The PCA dose was 0.25% bupivacaine with a 8 ml/h 10 ml bolus and 60-minute lockout. As part of the multimodal analgesia algorithm during the postoperative ward follow-up, the patient received paracetamol 1 g every 8 hours. Rescue analgesia was planned in the form of fentanyl 25 µg boluses in case of NRS ≥ 4 in the recovery unit, but this was not required. The catheter was removed at the end of the postoperative fourth day. The patient needed no pain relief other than paracetamol in the postoperative period and was satisfied with the quality of analgesia.

### 2.2. Case 2

A male patient aged 35 to 40 was taken to the operating room for laparoscopic liver surgery. General anesthesia was performed with 2 mg·kg^−1^ propofol, 1 μg·kg^−1^ fentanyl, and 0.6 mg·kg^−1^ rocuronium. Anesthesia was maintained by remifentanil infusion and desflurane. Laparoscopic metastasectomy was performed, with an operative time of 4 hours. After the surgery, unilateral EOI and bilateral TAP blocks were performed with the patient in the supine position. Blocks were performed under US guidance (GE, LOGIQ P9 R3, Seongnam-si, Republic of Korea) with a large bandwidth, multifrequency linear probe (4–12MHz), and a 22G, 50-mm, insulated facet type needle (Braun Sonoplex, Melsungen, Germany).

TAP block for each side involved 20 mL of 0.25% bupivacaine from the anterior axillary level between the internal oblique and transversus abdominis muscles. EOI block for the right side consisted of 20 mL of 0.25% bupivacaine from the sixth rib level between the external oblique and intercostal muscles. At the end of the surgery, 1 g paracetamol and tramadol 50 mg were administered iv. The patient received iv tramadol PCA (bolus dose 10 mg only, lockout 20 minutes). As part of the multimodal analgesia algorithm during the postoperative ward follow-up, the patient received paracetamol 1 g every 8 hours. Rescue analgesia was planned in the form of fentanyl 25 µg boluses in case of NRS ≥ 4 in the recovery unit. The patient needed a rescue dose only once in 60 minutes in the recovery unit. At the end of the postoperative 72nd hour, the use of tramadol PCA consisted of only 10 bolus doses. Total tramadol consumption was 100 mg, and the patient was satisfied with the quality of analgesia.

### 2.3. Case 3

A man aged 25 to 30 was taken to the operating room for laparoscopic bariatric surgery. General anesthesia was performed with 2 mg·kg^−1^ propofol, 1 μg·kg^−1^ fentanyl, and 0.6 mg·kg^−1^ rocuronium. Anesthesia was maintained by remifentanil infusion and 1 minimum alveolar concentration desflurane. A sleeve gastrectomy was performed, with an operative time of 3 hours 35 minutes. After the surgery, bilateral EOI block and bilateral RB were performed with the patient in the supine position. Blocks were performed under US guidance (GE, LOGIQ P9 R3, Seongnam-si, Republic of Korea) with a large bandwidth, multifrequency linear probe (4–12MHz), and a 22 G, 50-mm, insulated facet type needle (Braun Sonoplex, Melsungen, Germany). RB for each side consisted of 10 mL of 0.25% bupivacaine from the umbilicus level between the rectus abdominis muscle and transversalis fascia. EOI block for each side consisted of 20 mL of 0.25% bupivacaine from the sixth rib level between the external oblique and intercostal muscles. At the end of surgery, the patient was administered 1 g paracetamol and tramadol 50 mg iv. He received iv tramadol PCA (bolus dose 10 mg only, lockout 20 minutes). As part of the multimodal analgesia algorithm during the postoperative ward follow-up, the patients received paracetamol 1 g every 8 hours. Rescue analgesia was planned in the form of fentanyl 25 µg boluses in case of NRS ≥ 4 in the recovery unit. The patient required a rescue dose only twice in 60 minutes in the recovery unit. At the end of the postoperative 72nd hour, the use of tramadol PCA consisted of only 11 bolus doses. Total tramadol consumption was 110 mg, and the patient was satisfied with the quality of analgesia.

## 3. Discussion

US-guided abdominal wall blocks are generally easy to apply, reliable, and with low side-effect profiles. They represent serious alternatives to epidural and paravertebral blocks for abdominal and trunk analgesia, and can be applied during general anesthesia and are easily performed with the patient in the supine position. A different position is not usually required for these blocks. This constitutes a significant advantage. Other advantages include the areas of use, especially in case of contraindication of central neuraxial blocks, and that the technique can be learned and applied relatively easily.^[5,6]^ There are currently 2 possible mechanisms involved in analgesia in fascial plane blocks. The most important is a local effect on nociceptors and neurons in the fascial planes, the other being systemic vascular absorption of local anesthetics. Although there is no conclusive evidence in general, it is generally agreed that the effect of vascular absorption is unlikely. Consequently, the relative contributions of these local and systemic effects remain unclear.^[7]^ Diffusion is another important issue. During diffusion, fluid movement occurs, depending on the concentration difference. Diffusion between the fascial planes is unclear. The pores between the collagen fibers provide fascial permeability. Local anesthetic can follow 3 pathways in this fascial permeability. First, it can remain on the inter-fascial planes, alternatively, it can spread to the surrounding muscles and tissues through the pores, and finally it can spread to the systemic circulation via blood vessels. Diffusion of local anesthetic and possible systemic circulation, especially between the fascial planes, therefore seem to be the most likely effects.^[7–9]^ In addition, although the uncertainties regarding the mechanism of action remain, the effective results observed in the literature are an indication of further future development in fascial plane blocks.

Thoracic epidural analgesia remains important in open liver surgery. PROSPECT guidelines recommend bilateral subcostal TAP blocks with or without RBs as fascial plane blocks. Studies have shown that TAP block significantly reduces opioid consumption. EOI block is a modified block technique for upper midline and lateral abdominal wall analgesia. Elsharkawy et al^[10]^ demonstrated the potential mechanism of this technique with a cadaveric study reporting consistent staining of both lateral and anterior branches of intercostal nerves T7-T10. The fact that the EOI block used in the present case series reduces opioid consumption to almost zero may indicate that it will soon come very close to being the first choice as an opioid protective block.^[11]^

Fascial plane blocks are also used with epidural analgesia in laparoscopic liver surgery. However, the contribution of fascial plane blocks to visceral analgesia does not seem to be as successful as the somatic component. Kim et al^[12]^ described the erector spinae plane block as ineffective in their study of laparoscopic liver operations. There have been few studies of fascial plane blocks in laparoscopic liver surgery. We think that the effectiveness of the EOI block in the present case report may be a useful addition to the current literature. Ting Li et al^[13]^ reported that upper abdominal surgeries are more painful postoperatively than lower abdominal surgeries and require more opioids. Therefore, postoperative pain management is more important in upper abdominal surgeries. At this point, fascial plane blocks can be a savior.

Several studies have investigated fascial plane blocks in the context of bariatric surgery. TAP appears to be the most commonly used block. In their meta-analysis of TAP block in bariatric surgery, Tian et al^[14]^ reported a significant decrease in opioid consumption. However, there is still no consensus on the most effective TAP approach. There is not much information about EOV block in the literature. White et al^[15]^ reported 2 successful cases of 5EOI block. We agree that it is easy to apply, especially in obese patients. We argue that this is one of the most important advantages of the block. In addition, our case report on bariatric surgery is important as it is the first case in the literature. We think that the effective combination of EOI block and RB in the present case report will lead to new randomized controlled studies being conducted.

## 4. Conclusion

EOI block represents a promising approach to providing upper midline and lateral abdominal wall analgesia. Three different types of surgery are described in our case series. The EOI block yielded very successful results in different operations in which somatic and visceral pain components were present at different rates. A particularly valuable feature is that it offers 72 hours of analgesia, especially with a continuous catheter. EOI block may be important in obese patients in terms of ease of application. The application site can be easily visualized independently of body mass index and is easily accessible, and it also represents no disadvantage for obese patients under USG. From that perspective, we think that it will assume an important place in both bariatric and non-bariatric surgeries in obese patients. Work on identifying more effective fascial plane combinations and the emergence of new blocks will continue. However, we think that the EOI block will occupy an important place on that path.

## Acknowledgment

We are most grateful to Ms. Asli Baygül for her assistance with statistical analysis during the data collection.

## Author contributions

**Conceptualization:** Sami Kaan Coşarcan.

**Data curation:** Sami Kaan Coşarcan, Ömür Erçelen.

**Formal analysis:** Sami Kaan Coşarcan.

**Methodology:** Sami Kaan Coşarcan.

**Supervision:** Sami Kaan Coşarcan.

**Validation:** Sami Kaan Coşarcan.

**Visualization:** Sami Kaan Coşarcan.

**Writing – original draft:** Sami Kaan Coşarcan.

**Writing – review & editing:** Ömür Erçelen.
